# Rosai-Dorfman Disease: Rare Presentation as Isolated Mediastinal and Hilar Lymphadenopathy

**DOI:** 10.7759/cureus.2017

**Published:** 2018-01-02

**Authors:** Noman Lateef, Abdul Haseeb, Uzair K Ghori, Abubakar Tauseef, Mustafa Dawood, Syed M Hasan Kazmi

**Affiliations:** 1 Department of Medicine, Dow University of Health Sciences (DUHS), Karachi, Pakistan; 2 Pulmonology and Critical Care, Medical College of Wisconsin; 3 Medicine, Greater Baltimore Medical Center

**Keywords:** rosai-dorfman, lymphadenopathy, positron-emission tomography, lymph nodes, disease

## Abstract

Rosai-Dorfman disease (RDD) is a rare, benign, and predominantly nodal disease that most commonly presents as bilateral, painless cervical lymphadenopathy; although inguinal, axillary, mediastinal, and hilar lymphadenopathy has also been reported. Apart from nodal involvement, RDD has extranodal manifestations involving bone, soft tissue, and liver as well as constitutional symptoms of fever, night sweats, and weight loss, which make it reasonable to rule out the infectious, autoimmune, and malignant conditions as the differential diagnosis. We herein report a case of RDD affecting only the mediastinal and hilar region in an 83-year-old woman.

## Introduction

Rosai-Dorfman disease (RDD), also known as sinus histiocytosis with massive lymphadenopathy (SHML), is a rare and benign proliferative disorder of histiocytes [1] with approximately more than 750 reported cases. RDD is characterized by cervical lymphadenopathy in 95% of the cases, while some form of extranodal disease is seen in 30% to 43% of the patients [2]. There have been only nine reported cases of localized intrathoracic disease and among these cases, involvement only of the mediastinal-hilar region is very rarely reported [3]. Here we report a case of an 83-year-old woman with chief complaints of fatigue, malaise, and weakness, in whom RDD was diagnosed by transbronchial needle aspiration (TBNA) of mediastinal and hilar lymph nodes.

## Case presentation

An 83-year-old African-American woman with a past medical history of stage one breast cancer and Ig-A kappa multiple myeloma was referred to us for evaluation of avid lymphadenopathy on positron emission tomography (PET) scan. The patient had symptoms of fatigue, malaise, and weakness. She denied bone pain, weight loss, night sweats or any other active complaints. Her bone marrow biopsy showed increased plasmacytosis and hence a PET scan was ordered for further evaluation. The patient was on a combination of Velcade® (bortezomib) and dexamethasone.

**Figure 1:**
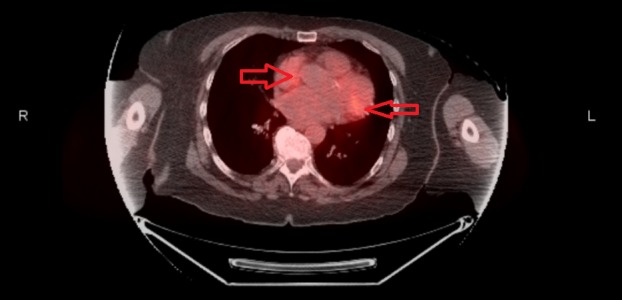
PET Scan Bilateral hyperintense metabolic lesions in the mediastinal region indicated by the red arrows. PET - positron emission tomography.

The physical examination was unremarkable except for poor inspiratory capacity. The laboratory findings were as follows: erythrocyte sedimentation rate (ESR) 35 mm/hr, calcium 10.4 mg/dL, and creatinine 1.2 mg/dL, while rest of the labs were normal. Her PET scan showed multiple intensely hypermetabolic mediastinal lymph nodes seen within the mediastinal region (Figure 1). There were no fluorodeoxyglucose (FDG) avid pulmonary nodules. The differential considerations included lymphoma, metastatic breast cancer, and sarcoidosis. A computed tomography-guided biopsy was planned for further evaluation but was unsuccessful. She was then scheduled for an endobronchial ultrasound (EBUS) to determine the etiology of mediastinal-hilar lymphadenopathy, and subsequently, transbronchial needle aspiration (TBNA) biopsies from lymph node stations 7, 11L, and 12R were taken.

**Figure 2:**
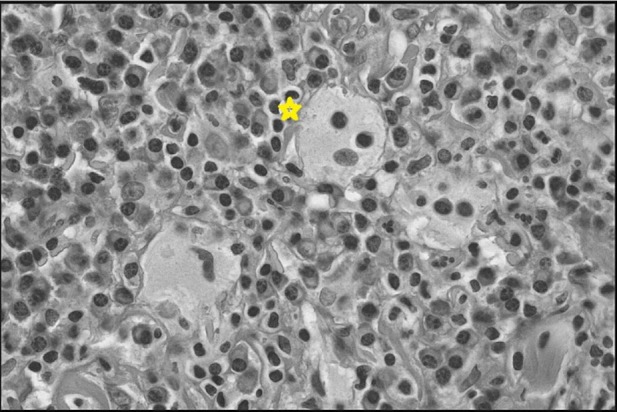
TBNA Biopsy Hematoxylin and eosin staining showing a foamy histiocyte with emperipolesis of lymphocytes (Asterisk (*)) with a background of mixed infiltrate of plasma cells, lymphocytes, and histiocytes. TBNA - transbronchial needle aspiration.

Histology showed that the specimen had foamy histiocytes with emperipolesis of lymphocytes with a mixed infiltrate of lymphocytes, plasma cells, histiocytes, and neutrophils in the background, but there was no evidence of granulomatous inflammation or malignant cells, which ruled out the differential diagnosis of sarcoidosis and metastatic breast cancer (Figure 2). Microscopic staining including Auramine O stain, Wade-Fite stain, PAS, and gram stains and culture for acid-fast bacilli, fungi, aerobic and anaerobic bacteria were also performed, but they all were negative. Immunohistochemistry showed that these cells stained positive for cluster of differentiation (CD) markers S-100, CD33, and CD68 but stained negative for CD1a. All these tests confirmed the diagnosis of Rosai-Dorfman disease. The patient received no treatment but remained under observation for monitoring of any further progress with follow-up every two months.

## Discussion

RDD was first reported in 1969 by pathologists Rosai and Dorfman as a non-neoplastic, proliferative, lymphohistiocytic, predominately nodal-based disease that is classified as an idiopathic proliferative disorder of histiocytes of reactive nature [1, 4]. The etiology and pathogenesis of RDD are uncertain but it has been postulated that it is associated with autoimmune and infectious causes. Human herpes virus 6 (HHV-6), Epstein-Barr virus (EBV), cytomegalovirus (CMV), Brucella, and Klebsiella have all been implicated but no one organism has been consistently isolated successfully [5]. Mean age at diagnosis for RDD is 20.6 years with a predilection for males and African population, but it can rarely occur in old age patients because of its benign course [2].

The most common clinical presentation of RDD is bilateral, massive, non-tender, painless cervical lymphadenopathy; present in around 90% of the cases, while other lymph nodes involved in decreasing order of frequency are inguinal, axillary, para-aortic, mediastinal, and hilar lymph nodes [6]. It is often associated with signs and symptoms either specific to the organ involved or generalized such as fever, night sweats, weight loss, fatigue, and sore throat [2]. Extranodal involvement as an isolated manifestation or with lymphadenopathy is seen in up to 43% of the patients with skin, soft tissue, upper respiratory tract, multifocal bone, eye, and retro-orbital tissue as the common sites. Among the laboratory findings, elevated ESR and polyclonal hypergammaglobulinemia is reported in 90% of patients. Leukocytosis with neutrophilia, normochromic normocytic anemia, and positive rheumatoid factor have also been reported, whereas, hemolytic anemia and eosinophilia are rare [7]. A definitive diagnosis of RDD can be made by histopathologic analysis; the presence of emperipolesis, i.e., engulfment of lymphocytes and erythrocytes by histiocyte-like cells that express S-100 is considered diagnostic of RDD. Apart from S-100 antigen positivity, immunohistochemical stains of RDD cells are also positive for CD68, CD163, α1-antichymotrypsin, α1-antitrypsin, fascin, and HAM-56 while CD1a is typically negative [8-9].

Our case presents an 83-year-old elderly woman who presented with subtle symptoms of fatigue, malaise, weakness, and hypermetabolic activity only of mediastinal and hilar lymph nodes on PET scan, all of which made the suspicion of recurrence of metastatic breast cancer, occult primary malignancy like lymphoma, and sarcoidosis as the likely possibilities. But the diagnosis of RDD was made by TBNA that effectively showed characteristic histology. On immunohistochemistry the most common positive cluster of differentiation (CD) marker is S-100; our patient was positive for S-100 as well as CD-68. Also, the ESR of the patient was elevated.

RDD has a variable prognosis with most reports indicating indolent and relatively good prognosis as the disease shows gradual resolution of lymphadenopathy in about 50% of the cases. In contrast, some fatal cases have also been reported. Extranodal disease especially of the liver and kidney, immunological abnormalities, and younger age render a poor prognosis. The disease can also be characterized by remission and exacerbation over months to years, hence long-term follow-up is necessary. Treatment if employed consists of corticosteroids that usually show good response, whereas, chemotherapy and radiotherapy are also used in some cases. Surgical resection is only indicated for massive and life or function-threatening disease [9].

Differentiation of RDD from malignant diseases such as lymphoma and occult metastatic disease solely on the basis of radiographic findings and PET scans can be difficult, as there can be a considerable overlap. Accordingly, in such cases, the diagnosis of RDD can be established on the grounds of characteristic histopathological findings via fine needle aspiration cytology (FNAC) or biopsy and benign clinical course of the disease.

To the best of our knowledge, there have been only two reported cases of RDD presenting only with mediastinal and hilar lymphadenopathy [10], hence further research is required to illustrate the characteristics of the disease and emphasize the point that the disease presentation can be as subtle in some cases as was in our patient.

## Conclusions

Although RDD is a rare and self-limiting disease, it is an important entity as it can imitate other fatal diseases. Consequently, it should be a consideration as an etiology of mediastinal and hilar lymphadenopathy, particularly in the picture of benign clinical course. And as such it should be differentiated from conditions like malignant lymphoma, occult metastatic disease, and sarcoidosis by relevant examinations and tests and avoiding other unnecessary investigations.
